# Guidelines (1988) for training in clinical laboratory management

**DOI:** 10.1155/S1463924689000234

**Published:** 1989

**Authors:** N. de Cediel, C. G. Fraser, A. Deom, L. Josefsson, H. G. J. Worth, O. Zinder

**Affiliations:** 1 15-47 Calle 49 Bogota Colombia; 2 Ninewells Hospital and Medical School Dundee UK; 3 Hôpital Cantonal Universitaire Geneva Switzerland; 4 University of Copenhagen Copenhagen Denmark; 5 King's Mill Hospital Sutton-in-Ashfield UK; 6 Rambam Medical Center Haifa Israel

## Abstract

Trainees in laboratory medicine must develop skills in laboratory management. Guidelines are detailed for laboratory staff in training, directors responsible for staff development and professional bodies wishing to generate material appropriate to their needs. The syllabus delineates the knowledge base required and includes laboratory planning and organization, control of operations, methodology and instrumentation, data management and statistics, financial management, clinical use of tests, communication, personnel management and training and research and development. Methods for achievement of the skills required are suggested. A bibliography of IFCC publications and other material is provided to assist in training in laboratory management.


Journal of Automatic Chemistry Vol. 11, No. 3 (May–June 1989), pp. 99–105

### Guidelines (1988) for training in clinical laboratory management

### International Federation of Clinical Chemistry (IFCC)†, Education Division and International Union of Pure and Applied Chemistry (IUPAC), Clinical Chemistry Division, Commission on Teaching of Clinical Chemistry

**Prepared for publication for the Education Division by:**
**N. de Cediel**
*15–47 Calle 49, Bogota, Colombia*
**C. G. Fraser**
*Ninewells Hospital and Medical School, Dundee, UK*
**A. Deom**
*Hôpital Cantonal Universitaire, Geneva, Switzerland*
**L. Josefsson**
*University of Copenhagen, Copenhagen, Denmark*
**H. G. J. Worth**
*King's Mill Hospital, Sutton-in-Ashfield, UK*
**O. Zinder**
*Rambam Medical Center, Haifa, Israel*

*Education Division members: O. Zinder (Israel), Chairman; H. G. J. Worth (UK), Secretary; N. de Cediel (Colombia); A. Deom (Switzerland); C. G. Fraser (UK); L. Josefsson (Denmark), representative of the International Union of Biochemistry.*
*Membership of the Teaching Commission during the period (1985–88) in which these Guidelines were prepared: O. Zinder (Israel), Chairman; H. G. J. Worth (UK), Secretary; Titular member, C. G. Fraser (UK); Associate members: M. A. Drodsdowsky (France); P. Garcia Webb (Austria); N. Montalbetti (Italy); C. J. Porter (Canada); B. Straus (Yugoslavia); V. N. Titov (USSR); R. Vihko (Finland); W. H. C. Walker (Canada); National Representatives: M. M. Abdel Kader (Egypt); J. Agneray (France); K. Bergstrom (Sweden); B. Christophersen (Norway); A. F. Delbruck (Denmark); H. A. Fritsche, Jr (USA); A. Gornall (Canada); A. G. Hadjivassiliou (Greece); T. Kanno (Japan); M. Nemeth Csoka (Hungary); P. Strom (Italy).*

*Trainees in laboratory medicine must develop skills in laboratory management. Guidelines are detailed for laboratory staff in training, directors responsible for staff development and professional bodies wishing to generate material appropriate to their needs. The syllabus delineates the knowledge base required and includes laboratory planning and organization, control of operations, methodology and instrumentation, data management and statistics, financial management, clinical use of tests, communication, personnel management and training and research and development. Methods for achievement of the skills required are suggested. A bibliography of IFCC publications and other material is provided to assist in training in laboratory management.*

† *The exclusive Copyright for all languages and countries is vested in the International Federation of Clinical Chemistry.*

### 1. Introduction

The scope of the disciplines that comprise laboratory medicine has expanded significantly in the last three decades. The range of quantities assayed and the variety and complexity of analytical techniques used have substantially increased. The turnaround time from the submission of a specimen to the receipt of a result has decreased and the performance characteristics of analytical procedures have continuously improved.

Simultaneously with these changes, the clinical use of results has also altered. Most results from patients in hospitals are used for management rather than as aids to diagnosis. Frequently, laboratory tests are performed prior to the actual clinical examination. In certain countries, the monitoring of apparently healthy individuals makes use of laboratory test results in preventative medicine. Hospital practices also have changed with significant ramifications for laboratory services; for example, more severely ill patients are being treated in specialist units such as intensive care, neonatal, coronary care and oncology units. The changing spectrum of disease, for example the growing number of patients with acquired immunodeficiency syndrome, also imposes new demands on laboratory services.

Hence laboratory medicine is not a static discipline and undoubtedly changes will continue to occur, probably ever more rapidly as time progresses. For this reason and because, with the current worldwide concern about the costs of health care, modifications and improvements in laboratory services will probably need to be introduced without significant new expenditure on staff or equipment, it is beholden upon trainees in laboratory medicine to develop adequate skills in *laboratory management*. This document is intended to serve as guidelines for training in this important area. It is suggested that the guidelines will be of value to laboratory staff learning management skills, directors of laboratories responsible for training staff in management and professional bodies wishing to generate guidelines appropriate to their national needs.

### 2. Scope

Although a considerable amount of knowledge about laboratory management is gained by experience, it is vital, particularly for those likely to become directors of

0142–0453/89 $3.00 © 1989 Taylor & Francis Ltd.

laboratories, to learn both theory and application in an ordered and systematic manner. The qualifications regarded as necessary prerequisites for appointment as a laboratory director differ from country to country but, irrespective of this, the syllabus detailed here is designed to fulfil the needs of both medical and science graduates.

Before commencing in-depth training in laboratory management, an adequate basic training must have been gained; ideally, for clinical chemists, the material detailed in the previous recommendations of the Committee/ Commission on a scheme for a 2-year postgraduate course in clinical chemistry should have been assimilated. It is particularly important that the science graduate is familiar with the clinical and interpretative aspects and that medical graduates have sufficient understanding of laboratory techniques before undergoing the training detailed in these guidelines.

### 3. Syllabus

The subject material to be included in the training programme is organized under the following headings:

3.1 Laboratory planning and organization.
3.2 Control of operations.
3.3 Methodology and instrumentation.
3.4 Data management and statistics.
3.5 Financial management.
3.6 Clinical use of tests.
3.7 Communication.
3.8 Personnel management and training.
3.9 Research and development.

### *3.1. Laboratory planning and organization*

It is essential that the individual responsible for laboratory management be able to plan and organize laboratory services and, as a necessary prerequisite, training should encompass the following:

3.1.1. Structure of health services in the country of the trainee, current national policy, priorities and resources.

3.1.2. Classification of laboratories (for example, primary, intermediate and specialist), interactions between types of laboratory, functions of the laboratory in diagnosis, management, screening, education and research and development.

3.1.3. Definition of workload and influencing factors, for example, local spectrum of diseases, expertise of clinical staff, availability of laboratory staff and equipment, type of population served—paediatric, adult, aged, chronic sick, acutely diseased, etc., assessment of workload using performance indicators, for example, unit values, number of requests and test per request ratio.

3.1.4. Strategies for organization of the laboratory; benefits and disadvantages of discretionary and profiling approaches, problems associated with biochemical screening, analytical equipment operated by non-laboratory personnel outside the laboratory (including local regulations, medical requirements, equipment and range of analyses available, training of analysts and quality assurance).

3.1.5. Organization of workflow, including the collection and transportation of specimens; identification of specimens and samples using colour codes, unique numbers, bar codes and other methods, distribution of specimens throughout the laboratory, work simplification techniques, referral of specimens to other laboratories.

3.1.6. Laboratory design; space requirements, optimum utilization of space, requirements for services such as electricity, gas and water, design requirements for handling radioisotopes, high-risk specimens and disposal of waste materials.

3.1.7. Organization of emergency services; advantages and disadvantages of dedicated laboratories, equipment and methods, turnaround times required for emergency tests, selection of the appropriate repertoire, strategies to monitor use and abuse of the emergency laboratory, setting of a hierarchy of priorities for test requests.

### *3.2. Control of operations*

All aspects of laboratory work should be monitored with the aim of always achieving the highest possible quality of performance. This implies that a *quality assurance* programme must be established, including internal quality control, participation in external quality assessment and a series of monitoring schemes specifically designed for other aspects of laboratory work, including the materials used, specimens submitted, staff morale and skills, reporting systems and turnaround times.

Training in laboratory management should therefore include the following:

3.2.1. Establishment of comprehensive internal quality control programmes, participation in quality assessment schemes, the availability of such schemes, analysis of the data generated in the assessment of imprecision, inaccuracy, linearity and other performance characteristics, the use of such data in method and instrument selection.

3.2.2. Quality control of specimens submitted and strategies to deal with inadequate specimens, quality control of materials and reagents, quality control of instrumentation including balances, water baths, incubators, refrigerators, spectrometers, automated analysers, isotope counters, pipettes, diluters, dispensers, etc., quality control of data handling and calculation facilities, monitoring of the performance of individual members of staff, quality control of reporting systems and turnaround times.

3.2.3. Preparation and use of laboratory procedure manuals as both educational material and a means of ensuring that all methodology is maintained at a constant high standard.

3.2.4. Setting of desirable standards of analytical performance, strategies for the improvement of laboratory performance with existing staff, methods, instruments and resources.

*3.3. Methodology and instrumentation*

Correct selection and use of methodology and instrumentation are of vital importance if the laboratory is to play a full role in the provision of optimal health care; adequate training therefore must be provided in the following:

3.3.1. Preparation of ideal specifications for methods, instrumentation and reagent kit sets in order to facilitate selection, assessment and evaluation of methods, instruments and reagent kit sets, introduction of new methods into regular use in the laboratory.

3.3.2. Purchase of equipment, preparation of documentation required for funding and purchase, negotiation of warranty and service agreements.

3.3.3. Maintenance of equipment, preparation of equipment usage and maintenance logs.

*3.4. Data management and statistics*

The optimal use of laboratory data is not always achieved; a knowledge of appropriate techniques for data handling and interpretation is essential for the laboratory manager. Moreover, the ever increasing use of computers requires knowlege of their applications and limitations. A working knowledge of statistical techniques and their correct application is also required.

Training therefore should include the following:

3.4.1. Units, use of numerical data in management, the fundamentals of computers, mainframe, mini- and microcomputers, networks, applications of computers, on-line acquisition of data from instruments, preparation of work lists, patient databases and reports, use of computers in quality control and assessment, expert systems, system design, availability of commercial and other laboratory computer systems, data storage, retrieval and confidentiality.

3.4.2. Laboratory calculations, curve-fitting routines, data handling for radioimmunoassay and other ligand assays.

3.4.3. Statistics including common parametric and non-parametric techniques; mode, median and mean, range, standard deviation, variance, linear regression, correlation, and probability, Deming's method, *t*-tests, *F*-test, Wilcoxon test, simple analysis of variance.

*3.5. Financial management*

In addition to gaining a basic understanding of budgetting systems, especially those adopted locally, an awareness of the following must be gained:

3.5.1. Costing of laboratory tests, division of expenditure into fixed (staff, instrumentation, etc.) and variable (consumables, reagents, etc.) costs, patient billing and/or clinical (management) budgetting (if appropriate), virement, methods of obtaining additional resources.

3.5.2. Costs of consumables, advantages and disadvantages of bulk purchase and standing orders, costs of service contracts, amortization of equipment costs and advantages and disadvantages of hire agreements.

3.5.3. Budgetary planning for future activities, presentation of budgets, projected expenditure on method development, instrument purchase, expansion and/or reorganization of services.

*3.6. Clinical use of tests*

It is of vital importance that senior laboratory staff are able to advise the clinician not only on the selection of the most appropriate tests and the interpretation of results, but also on the nosological characteristics of the tests. Moreover, the ability to discuss the introduction of new tests and the phasing out of obsolete tests with clinicians must be gained, as must the knowledge to be able to develop efficient and effective strategies for the use of the laboratory. Training should therefore cover the following topics:

3.6.1. The theory of reference values, selection of reference individuals, statistical approaches to generation of reference values, the endogenous, exogenous, ethnic, genetic and laboratory factors that affect reference values and strategies for objective comparison of observed values with reference values, biological variability and the uses of biological variation data.

3.6.2. The uses of knowledge of the nosological sensitivity and specificity and predictive value of tests, receiver-operating characteristic curves and likelihood ratios, objective analysis of clinical literature on laboratory test use.

3.6.3. Strategies to modify the requesting behaviour of clinicians (including rationing, education, budget incentives and development of protocols for investigation), introduction of new tests and elimination of obsolete tests.

3.6.4. Education of medical studies, training of junior clinical staff, establishment of joint clinical/laboratory educational activities, preparation of laboratory case reports.

*3.7 Communication*

Inter-personal communication is vital and training should not only encompass communication with clinical staff on an individual level concerning the matters dealt with in Section 3.6 but also be on a broader basis, to include:

3.7.1. Communication between staff within the laboratory.

3.7.2. Communication with laboratory users through laboratory bulletins, request forms, reports and laboratory handbooks.

3.7.3. The advantages and limitations of the various styles of request forms, styles of single or cumulative reports and laboratory data filing systems.

3.7.4. Communication with administrative and laboratory staff, preparation of reports and memoranda, committee structures and procedures, roles of Chairman, Secretary and other members, taking of minutes and preparations of agenda.

*3.8. Personnel management and training*

The individual responsible for laboratory management must be adequately trained in dealing with the most important laboratory resource—the laboratory staff—and therefore training should encompass the following:

3.8.1. Laboratory staff structures, staff selection procedures, preparation of job descriptions, setting of responsibilities and chains of command, promotional procedures, disciplinary and grievance procedures, legal conditions of service, requirements for licensure and certification.

3.8.2. Evaluation of individual members of staff, assignment of functions and responsibilities.

3.8.3. Training and education of staff according to their level, assessment of capabilities, career needs and aspirations, development of in-house training programmes and the evaluation of these, liaison with external educational institutions and professional bodies.

3.8.4. Laboratory safety including fire precautions, handling of potentially hazardous specimens and chemicals, disposal of wastes, accident reporting, awareness of local and national requirements of legislation.

*3.9. Research and development*

Although most individuals who are in the final stages of training for a career in laboratory management will have performed some research and development work, it is essential that adequate skills be gained in the following:

3.9.1. The ability to develop improvements in methods and techniques, to evaluate proposals for both laboratory-based and clinical research projects and to evaluate published work critically.

3.9.2. Analysis and documentation of results obtained through research and development, presentation of results in lectures, seminars and workshops, oral and poster presentations at conferences, congresses and meetings and the preparation of scientific papers.

3.9.3. Preparation of requests for grant funding, development of proposals for joint research projects, role of committees on ethnics of research.

3.9.4. Supervision of junior staff and students in the day-to-day performance of research and development projects.

### 4. Achievement of skills

As stated earlier, a considerable amount of knowledge on laboratory management will be accumulated through experience; however, it is advisable, if possible, for trainees to attend local or national courses that deal with the more general issues of management, for example, personnel and finance.

These courses often have participants from a number of disciplines which enhances their value.

Visits to other laboratories should be undertaken and a spectrum of types and sizes should be studied during the training period in order to assess both the common and different management problems and view the different approaches to solving problems. Indeed, attainment of the skills required may necessitate the trainee being formally employed in different laboratories or being seconded for appropriate lengths of time to laboratories of different types.

Ideally, much of the training in laboratory management should be performed in a large tertiary care teaching hospital laboratory where a large repertoire of tests are performed on a wide variety of specimens from patients with a broad range of clinical conditions. This will facilitate review and tutorial sessions with a number of senior members of staff with different qualifications, backgrounds, interests and experience. Moreover, in such laboratories, there is likely to be a cohort of individuals in training which facilitates learning by, for example, the setting up of discussion groups, interactive solution of problem-solving exercises and simple peer pressure. In addition, it is easier in these situations gradually to give the trainee increasing responsibilities and management functions.

It is considered unlikely that a full-time didactic course in laboratory management will be a satisfactory educational vehicle. A part-time course of, for example, one evening or day per week over 1 year would have advantages; the number of participants should be limited to ensure educational effectiveness.

An important component of the advocated training in laboratory management is the performance of relevant project work. Circumscribed projects, for example, on the selection of a new instrument, preparation of a budget for a section of the laboratory, assessment of a new clinical test in collaboration with a clinician, would provide useful exercises during training. A summary project, of potential benefit to the laboratory of the trainee, could be a study reviewing the management of the laboratory in which the trainee is employed.

It is most important for the head of the laboratory to ensure the availability of adequate resources for training in laboratory management, to encourage the development of managerial skills and to involve the trainee (even as an observer) in the real decision-making processes of the laboratory.

Laboratory medicine is continuously and rapidly evolving, and therefore the syllabus detailed in these guidelines should not be regarded as inflexible, but should be modified as changes in practice occur. Moreover, in different countries, there are diverse approaches to laboratory management of laboratories and these guidelines should be modified locally as deemed necessary; professional bodies are considered to be ideal groups to perform such changes.

### 5. Bibliography

There are no texts which satisfactorily cover all of the material outlined in the syllabus. The list of books, papers and other sources given below is aimed (i) to facilitate curriculum design, development and implementation by those responsible for training individuals in management and (ii) to aid individuals who are undertaking training in laboratory management. The bibliography is divided into two sections: (i) recommendations and other publications emanating from the IFCC and (ii) other material judged to be of value and relevance. The content is mainly concerned with clinical chemistry.

Much valuable information on many of the topics detailed in this syllabus can be found in the many excellent and widely used text books, such as Tietz, N.W. (Ed.), *Fundamentals of Clinical Chemistry*, 3rd edn (Saunders, Philadelphia, 1985); this and other similar texts should be familiar to those embarking upon in-depth training in laboratory management and are therefore not included in the bibliography.

### *5.1. IFCC Publications*

#### *5.1.1 Quality control*

1. Buttner, J., Borth, R., Boutwell, J. H., Broughton, P. M. G. and Bowyer, R. C., Approved recommendations (1978) on quality control in clinical chemistry. Part 1. General principles and terminology, *Journal of Clinical Chemistry and Clinical Biochemistry*, **18** (1980), 69–77; *Clinica Chimica Acta*, **98** (1979), 129F–143F.
2. Buttner, J., Borth, R., Boutwell, J. H., Broughton, P. M. G. and Bowyer, R. C., Approved recommendations (1979) on quality control in clinical chemistry. Part 3. Calibration and control materials, *Journal of Clinical Chemistry and Clinical Biochemistry*, **18** (1980), 855–860; *Clinica Chimica Acta,* **109** (1981), 105F–114F.
3. Buttner, J., Borth, R., Boutwell, J. H., Broughton, P. M. G. and Bowyer, R. C., Approved recommendations (1983) on quality control in clinical chemistry. Part 4. Internal quality control, *Journal of Clinical Chemistry and Clinical Biochemistry*, **21** (1983), 877–884.
4. Buttner, J., Borth, R., Boutwell, J. H., Broughton, P. M. G. and Bowyer, R. C., Approved recommendations (1983) on quality control in clinical chemistry. Part 5. External quality control, *Journal of Clinical Chemistry and Clinical Biochemistry*, **21** (1983), 885–892.
5. Buttner, J., Borth, R., Boutwell, J. H., Broughton, P. M. G., and Bowyer, R. C., Approved recommendations (1979) on quality control in clinical chemistry. Part 6. Quality requirements from the point of view of health care, *Journal of Clinical Chemistry and Clinical Biochemistry*, **18** (1980) 861–866; *Clinica Chimica Acta*, **109** (1981), 115F–124F.
6. Fraser, C. G., Geary, T. D. and Worth, H. G. J., Guidelines (1986) for the preparation of laboratory procedure manuals, *Journal of Clinical Chemistry and Clinical Biochemistry*, **26** (1988), 415–419.

#### *5.1.2. Methodology and instrumentation*

7. Buttner, J., Borth, R., Boutwell, J. H., Broughton, P. M. G. and Bowyer, R. C., Approved recommendations (1978) on quality control in clinical chemistry. Part 2. Assessment of analytical methods for routine use, *Journal of Clinical Chemistry and Clinical Biochemistry*, **18** (1980), 78–88; *Clinica Chimica Acta* **98**, (1979), 145F–162F.
8. Logan, J. E., Revised recommendations (1983) on evaluation of diagnostic kits. Part 1. Recommendations for specifications on labelling of clinical laboratory materials, *Journal of Clinical Chemistry and Clinical Biochemistry*, **21** (1983), 893–898; *Clinica Chimica Acta*, **137**, (1984), 371F–379F; *Clinical Chemistry Newsletter*, **5** (1985), 81–86.
9. Logan, J. E., revised recommendations (1983) on evaluation of diagnostic kits. Part 2. Guidelines for the evaluation of clinical chemistry kits. *Journal of Clinical Chemistry and Clinical Biochemistry*, **21** (1983), 899–902; *Clinica Chimica Acta*, **137**, (1984) 381F–386F; *Clinical Chemistry Newsletter* **5** (1985), 87–90.
10. Logan, J. E., Bayse, D. D., Koedam, J. C., Mather, A. and Wilding, P., IFCC/WHO principles and recommendations on evaluation of diagnostic reagent sets used in health laboratories with limited resources. Part 3. Selection and evaluation using reference materials. General considerations, *Journal of Clinical Chemistry and Clinical Biochemistry*, **22** (1984), 573–582.
11. Okuda, K., Provisional guidelines (1981) for listing specifications of clinical chemistry analysers, *Journal of Clinical Chemistry and Clinical Biochemistry*, **18** (1980), 947–951; *Clinica Chimica Acta*, **119** (1982), 351F–362F; *Clinical Biochemistry*, **13** (1980), 244–248.
12. Donohoe, G. A., Geary, T. D. and Jennings, R. D. Bibliography on evaluation of instrumentation in clinical chemistry. *Journal of Clinical Chemistry and Clinical Biochemistry*, **20** (1982), 931–945; *Clinica Chimica Acta*, **127** (1983), 425F–439F, *Clinical Chemistry Newsletter*, **3** (1983), 3–13.

#### *5.1.3 Units*

13. Dybkaer, R., Approved recommendations (1978). Quantities and units in clinical chemistry. *Journal of Clinical Chemistry and Clinical Biochemistry*, **17** (1979), 807–821;

*Clinica Chimica Acta*, **96** (1979), 155F–183F; *Pure and Applied Chemistry*, **51**, (1979), 2451–2479.

14. Dybkaer, R., Approved recommendations (1978). List of quantities in clinical chemistry. *Journal of Clinical Chemistry and Clinical Biochemistry*, **17** (1979), 822–835; *Clinica Chimica Acta*, **96** (1979), 185F–204F; *Pure and Applied Chemistry*, **51** (1979), 2481–2509.
15. Lehmann, P., Worth, H. and Zinder, O., Clinical chemists convert to the mole, *Chemistry International*, **10** (1988), 52–57.

*5.1.4 Reference values*

16. Solberg, H. E., Approved recommendations (1986) on the theory of reference values. Part 1. The concept of reference values, *Journal of Clinical Chemistry and Clinical Biochemistry*, **25** (1987), 337–342; *Clinica Chimica Acta*, **165** (1987), 111–118; *Annals of Biological Chemistry*, **45** (1987), 237–241.
17. PetitClerc, C. and Solberg, H. E., Approved recommendations (1987) on the theory of reference values. Part 2. Selection of individuals for the production of reference values. *Journal of Clinical Chemistry and Clinical Biochemistry*, **25** (1987), 639–644; *Clinica Chimica Acta*, **170**, (1987), S1–S12.
18. Solberg, H. E. and PetitClerc, C., Approved recommendations (1988) on the theory of reference values. Part 3. Preparation of individuals and collection of specimens for the production of reference values. *Journal of Clinical Chemistry and Clinical Biochemistry*, **26** (1988), 593–598.
19. Solberg, H. E., Approved recommendations (1987) on the theory of reference values. Part 5. Statistical treatment of collected reference values. Determination of reference limits. *Journal of Clinical Chemistry and Clinical Biochemistry*, **25** (1987), 645–656; *Clinica Chimica Acta*, **170** (1987), S13–S32.
20. Dybkaer, R. and Solberg, H. E., Approved recommendations on the theory of reference values. Part 6. Presentation of observed values related to reference values. *Journal of Clinical Chemistry and Clinical Biochemistry*, **25** (1987), 657–662; *Clinica Chimica Acta*, **170**, (1987) S33–S42.

*5.1.5. Communication*

21. Fraser, C. G., deCediel, N., Porter, C. J., Schwartz, M. K., Worth, H. G. J. and Zinder, O., Guidelines (1985) for clinical chemists for effective communication of clinical chemistry laboratory data, *Journal of Clinical Chemistry and Clinical Biochemistry*, **23** (1985), 891–897.

*5.1.6 Training*

22. Porter, C. J. and Curnow, D. H., A scheme for a two year postgraduate course in clinical chemistry. *Journal of Clinical Chemistry and Clinical Biochemistry*, **21** (1983), 185–191; *Clinica Chimica Acta*, **131** (1983), 351F–359F; *Pure and Applied Chemistry*, **55** (1983), 557–564.
23. Worth, H. G. J., A basic education and training framework for medical laboratory technicians in clinical chemistry, *Journal of Clinical Chemistry and Clinical Biochemistry*, **22** (1984), 497–501; *Clinica Chimica Acta*, **141** (1984), 305F–311F; *Clinical Chemistry Newsletter*, **5** (1985), 97–101; *Pure and Applied Chemistry*, **56** (1984), 1505–1510.
24. Fraser, C. G., Zinder, O., deCediel, N., Porter, C. J., Schwartz, M. K. and Worth, H. G. J., Guidelines (1985) for teaching of clinical chemistry to medical students. *Journal of Clinical Chemistry and Clinical Biochemistry*, **23** (1985), 697–703; *Clinical Chemistry Newsletter*, **5** (1985), 102–108.
25. Schwartz, M. K., deCediel, N., Curnow, D. H., Fraser, C. G., Porter, C. J., Worth, H. G. J. and Zinder, O., Definition of the terms certification, licensure and accreditation in clinical chemistry, *Journal of Clinical Chemistry and Clinical Biochemistry*, **23** (1985), 899–901.
26. Pannall, P. R., Dennis, P. M., Farrance, I. and Garcia-Webb, P., Guidelines for the training of medical graduates in clinical chemistry, *Journal of Clinical Chemistry and Clinical Biochemistry*, **26** (1988), 585–591.

*5.1.7 Safety*

27. Bonini, P. A., Safety in clinical laboratories, *IFCC News*, **37** (1984), 10–11.

*5.2. Other sources*

*5.2.1 General planning and organization*

28. WHO, *Laboratory Services at Primary Health Care Level*, Lab/79.1 (WHO, Geneva, 1979).
29. WHO, Working Group on Assessment of Clinical Technologies, (i) *Identification of Essential Clinical Chemical and Haematological Tests in Intermediate Hospital Laboratories*, Lab/86.2; (ii) *Methods Recommended for Essential Clinical Chemical and Haematological Tests for Intermediate Hospital Laboratories*, Lab/86.3 (WHO, Geneva, 1986).
30. Benson, E. S. and Rubin, M. (Eds), *Logic and Economics of Clinical Laboratory Use* (Elsevier, New York, 1978).
31. Noe, D. A., *The Logic of Laboratory Medicine*, (Urban and Schwartzenburg, Baltimore, 1985).
32. Lundberg, G. D. (Ed.), *Using the Clinical Laboratory in Medical Decision-Making* (ASCP, Chicago, 1983).
33. Speicher, C. E., and Smith, J. W., *Choosing Effective Laboratory Tests* (Saunders, Philadelphia, 1983).
34. Fraser, C. G., *Interpretation of Clinical Chemistry Laboratory Data* (Blackwell, Oxford, 1983).
35. Bermes, E. W. (Ed.), *The Clinical Laboratory in the New Era: Quality, Cost and Diagnostic Demands* (AACC, Washington, 1985).
36. Marks, V. and Alberti, K. G. M. M. (Eds), *Clinical Chemistry Nearer the Patient* (Churchill Livingstone, Edinburgh, 1985).
37. *Canadian Schedule of Unit Values for Clinical Laboratory Procedures* (Statistics Canada, Ottawa, 1977).
38. Slockbower, J. M. and Blumenfeld, T. A., (Eds), *Collection and Handling of Laboratory Specimens: a Practical Guide*, (Lippincott, Philadelphia, 1983).
39. Barnett, R. N., McIver, D. D. and Gorton, W., The medical usefulness of stat tests, *American Journal of Clinical Pathology*, **69** (1978), 520–524.

*5.2.2 Control of operations*

40. Whitehead, T. P., *Quality Control in Clinical Chemistry*, (Wiley, Chichester, 1977).
41. Bruce, A. W., *Basic Quality Assurance and Quality Control in the Clinical Laboratory*, (Little, Brown and Co., Boston, 1984).
42. Fraser, C. G., Desirable performance standards for clinical chemistry tests, *Advances in Clinical Chemistry*, **23** (1983), 299–339.
43. Jeffcoate, S. L., *Efficiency and Effectiveness in the Endocrine Laboratory*, (Academic Press, London, 1981).

*5.2.3. Methodology and instrumentation*

44. Lloyd, P. H., A scheme for the evaluation of diagnostic kits, *Annals of Clinical Biochemistry*, **15** (1978), 136–145.
45. Broughton, P. M. G., Gowelock, A. H., McCormack, J. J. and Neill, D. W., A revised scheme for the evaluation of automatic instruments for use in clinical chemistry, *Annals of Clinical Biochemistry*, **11** (1974), 207–218.
46. White, G. H. and Fraser, C. G., The evaluation kit for clinical chemistry: a practical guide for the evaluation of methods, instrumentation and reagent kits, *Journal of Automatic Chemistry*, **6** (1984), 122–144.

47. Clark, I., Peters, M. and Broughton, P. M. G., Evaluation of the computing aspects of automatic analysers, *Annals of Clinical Biochemistry*, **23** (1986), 585–589.
48. Fraser, C. G. and Singer, R., Better laboratory evaluations of instruments and kits are required, *Clinical Chemistry*, **31** (1985), 667–670.

*5.2.4 Data management and statistics*

49. Barnett, R. N., *Clinical Laboratory Statistics*, 2nd edn (Little, Brown and Co., Boston, 1974).
50. Swinscow, T. D. V., *Statistics at Square One*, 5th Edn (British Medical Association, London, 1980).
51. De Bats, A. and O'Meara, J. (Ed), *A Guide to Data Processing in Clinical Laboratories.* (Association of Clinical Biochemists, London, 1983).

*5.2.5. Financial management*

52. Worth, H. G. J., Relative costing of analytical systems. *Journal of Automatic Chemistry*, **2** (1980), 125–133.
53. Broughton, P. M. G. and Hogan, T. C., A new approach to the costing of clinical laboratory tests, *Annals of Clinical Biochemistry*, **18** (1981), 330–342.
54. Stilwell, J. A., The costs of a clinical chemistry laboratory, *Journal of Clinical Pathology*, **34** (1981), 589–594.
55. Broughton, P. M. G. and Woodford, F. P., Benefits of costing in the clinical laboratory, *Journal of Clinical Pathology*, **26** (1983), 1028–1035.
56. Tarbit, I. F., Costing clinical biochemistry services as part of an operational management system, *Journal of Clinical Pathology*, **39** (1986), 817—827.
57. WHO, *Assessment of Benefits and Costs of Clinical Laboratory testing*, Lab/84.5. (WHO, Geneva, 1984).

*5.2.6 Clinical use of tests*

58. Grassbeck, R. and Alstrom, T. (Eds), *Reference Values in Laboratory Medicine. The Current State of the Art*, (Wiley, Chichester, 1981).
59. Meites, S. (Ed.), *Pediatric Clinical Chemistry*, 2nd edn (AACC, Washington, 1981).
60. Galen, R. S. and Gambino, S. R. *Beyond Normality: the Predictive Value and Efficiency of Medical Diagnoses*, (Wiley, New York, 1975).
61. Zweig, M. H. and Robertson, E. A., Why we need better test evaluations. *Clinical Chemistry*, **28** (1982), 1272–1276.
62. Fraser, C. G. and Woodford, F. P., Strategies to modify the test-requesting patterns of clinicians, *Annals of Clinical Biochemistry*, **24** (1987), 223–231.

*5.2.7 Communication*

63. Lundberg, G. D., Laboratory request forms (menus) that guide and teach, *Journal of the American Medical Association*, **249** (1983), 3075.
64. Burns, E. L., Hanson, D. J., Schoen, I., Barnett, R. N., Minckler, T. and Winter, S., Communication of laboratory data to the clinician, *American Journal of Clinical Pathology*, **61** (1975), 900–903.

*5.2.8 Training*

65. Zinder, O., deCediel, N., Curnow, D. H., Fraser, C. G., Porter, C. J., and Worth, H. G. J., The education of the clinical chemist, *Clinical Chemistry Newsletter*, **1/2** (1987), 23–29.

*5.2.9 Research and development*

66. Reigelman, R. K., *Studying a Study and Testing a Test. How to Read the Medical Literature*, (Little, Brown and Co., Boston, 1981).

